# TGF-β receptor inhibitor LY2109761 enhances the radiosensitivity of gastric cancer by inactivating the TGF-β/SMAD4 signaling pathway

**DOI:** 10.18632/aging.102329

**Published:** 2019-10-19

**Authors:** Tian Yang, Tianhe Huang, Dongdong Zhang, Miao Wang, Balu Wu, Yufeng Shang, Safat Sattar, Lu Ding, Yin Liu, Hongqiang Jiang, Yuxing Liang, Fuling Zhou, Yongchang Wei

**Affiliations:** 1 Department of Radiation and Medical Oncology, Zhongnan Hospital of Wuhan University, Wuhan 430071, China; 2 Department of Clinical Oncology, The First Affiliated Hospital of Xi’an Jiaotong University, Xi’an 710061, China; 3 Department of Biological Sciences, Boler-Parseghian Center for Rare and Neglected Diseases, Harper Cancer Research Institute, University of Notre Dame, Notre Dame, IN 46556, USA; 4 Department of Hematology, Zhongnan Hospital of Wuhan University, Wuhan 430071, China

**Keywords:** transforming growth factor-β (TGF-β), gastric cancer (GC), radioresistance, tumor microenvironment, overall survival (OS)

## Abstract

Radiotherapy is used to treat gastric cancer (GC); however, radioresistance challenges the clinical outcomes of GC, and the mechanisms of radioresistance in GC remain poorly understood. Here, we report that the TGF-β receptor inhibitor, LY2109761 (LY), is a potential radiosensitizer both in vitro and in vivo. As per the Cancer Genome Atlas database, TGF-β overexpression is significantly related to poor overall survival in GC patients. We demonstrated that the TGF-β/SMAD4 signaling pathway was activated in both radioresistant GC cells and radioresistant GC patients. As a TGF-β receptor inhibitor, LY can enhance the activities of irradiation by inhibiting cell proliferation, decreasing clonogenicity and increasing apoptosis. Moreover, LY attenuated the radiation-induced migration and invasion, epithelial-mesenchymal transition (EMT), inflammatory factor activation, immunosuppression, and cancer stem cell characteristics of GC cells, thus leading to radiosensitization of the GC cells. We confirmed that LY reduced tumor growth, inhibited TGF-β/SMAD4 pathway activation and reversed irradiation-induced EMT in a tumor xenograft model. Our findings indicate that the novel TGF-β receptor inhibitor, LY, increases GC radiosensitivity by directly regulating the TGF-β/SMAD4 signaling pathway. These findings provide new insight for radiotherapy in GC patients.

## INTRODUCTION

Gastric cancer (GC) is one of the most common and malignant tumors of the digestive system [1] and is difficult to diagnose at an early stage [2]. Radiotherapy is a standard treatment for patients with advanced GC [3]. However, patients with GC receive limited benefit from radiotherapy, and most develop tolerance to radiotherapy [4]. Although numerous genes have been demonstrated to be related to radiosensitivity in different cancers [5, 6], the clear mechanisms of radioresistance in GC remain unknown. Therefore, the mechanisms of GC radioresistance must be clarified, and new radiosensitizers must be developed to treat GC.

The main principle of radiotherapy is to induce cell death by damaging or rupturing the DNA double helix with ionizing radiation (IR) such as X-rays, neutrons, α-rays, β-rays, and γ-rays [7, 8]. However, while killing tumor cells, radiation also influences the tumor microenvironment containing the tumor cells [9]. The tumor microenvironment consists of tumor cells, fibroblasts, vascular cells, immune cells and the extracellular matrix. Several studies have shown that interactions between multiple cells in the tumor microenvironment are the main cause of treatment tolerance to radiotherapy [10]. The epithelial-mesenchymal transition (EMT) contributes to radioresistance in many malignant tumors by dysregulating the EMT markers, E-cadherin and N-cadherin [11]. Inflammatory reactions are also positively correlated with tumor radioresistance. The inflammatory cytokines, interleukin (IL)1, IL2 and IL6, in the tumor microenvironment are activated after irradiation treatment in many tumors [12]. Moreover, the radiotherapy-mediated immunosuppression in the tumor microenvironment has been intensively investigated in many studies [13, 14]. In addition, cancer stem cells (CSC) in the tumor microenvironment play important roles in radioresistance and affect tumor development, invasiveness and metastatic dissemination. The TGF-β family includes polypeptides play important roles in regulating tumor cells, tumor-associated fibroblasts and immunorelated cells in the tumor microenvironment [15, 16]. TGF-β1, -β2 and -β3 cytokines promote the progression of several cancers, including GC, by binding the TGF-β-type receptor (TβR) [17]. Research has shown that TGF-β expression is upregulated in the tumor microenvironment after radiotherapy [18, 19]. The mothers against decapentaplegic protein (SMAD4) in Drosophila is a member of the SMAD family and is activated by transmembrane serine-threonine receptor kinases in response to TGF-β signaling. Research has shown that TGF-β/SMAD4 signaling pathway activation promotes EMT, and the TGF-β signaling pathway modulates immune functions and activates inflammatory factors in diverse malignant neoplasms [20, 21]. Thus, GC initiation and progression promoted by the TGF-β/SMAD4 pathway may be reversed by TGF-β inhibitors. LY2109761 (LY) is a novel selective TGF-β receptor type I/II inhibitor that completely inhibits TGF-β-induced SMAD2 phosphorylation and exhibits antitumor effects in various tumor models, such as glioblastoma [22], oral squamous cell carcinoma [23] and prostate cancer [24]. LY may thereby play an important role in GC therapy by inactivating the TGF-β/SMAD4 signaling pathway.

Here, we examined the activities of the TGF-β/SMAD4 pathway in GC radioresistance and the potential role of TGF-β-receptor inhibitors in GC radiosensitivity in vivo and in vitro.

## RESULTS

### High levels of TGF-β were associated with poor overall survival (OS) of GC patients

**Figure 1:**
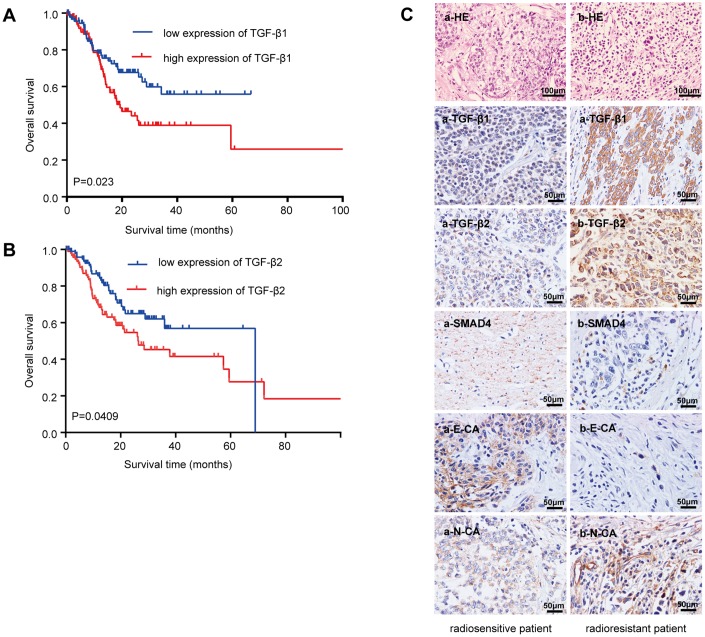
**Relationship between the TGF-β expressions with the overall survival and radioresistance in GC patients.** (**A**) Kaplan-Meier survival curves for high and low mRNA TGF-β1 expression level in GC patients. (**B**) Kaplan-Meier survival curves for high and low mRNA TGF-β2 expression level in GC patients. (**C**) Immunohistochemical analysis: representative immunohistochemistry images of the TGF-β1, TGF-β2, SMAD4, E-CA and N-CA expressions in biopsy samples of GC patients. (**a**) Specimen from a patient whose tumor sample showed a complete response to radiotherapy (radiosensitive GC patient). (**b**) Specimen from a patient whose tumor sample showed no change after radiotherapy (radioresistant GC patient). Original magnification, ×400. *P<*0.05.

Analyzing the Cancer Genome Atlas database (Figure 1A) showed that GC patients with low TGF-β1 expression generally had a prolonged OS (median survival time undefined) and that upregulated TGF-β1 was significantly correlated with poor OS (median survival time: 19.32 months, n=203, *P=*0.023). Figure 1B shows that GC patients with low TGF-β2 expression had a prolonged OS (median survival time 68.99 months) compared with that of patients with high TGF-β2 expression (median survival time 26.45 months, n=205, *P=*0.0409). These data showed that TGF-β may be a better prognostic marker than grading or tumor necrosis metastasis (TNM) staging (not significantly related to GC patient prognoses; Supplementary Figure 1A).

### Dysregulation of TGF-β and EMT markers in GC patients with radioresistance

Twenty-four cancer tissue samples were harvested from GC patients before radiotherapy (diagnostic biopsies). Computed tomography images from before and two months after radiotherapy were reviewed to examine the radiosensitivity of these samples. Sixteen specimens responded completely to radiotherapy (radiosensitive tissue), while 8 were nonreactive after radiotherapy (radioresistant tissue). Table 1 lists the patients’ characteristics. TGF-β1 and TGF-β2 were strongly upregulated in radioresistant GC patients compared with those in radiosensitive patients (Figure 1C). The downstream molecule, SMAD4, was downregulated, and EMT makers were dysregulated in radioresistant GC patients (E-cadherin was downregulated, and N-cadherin was upregulated). The staining indices differed statistically (Supplementary Figure 1B).

**Table 1:** Clinical characteristics of patients.

**Characteristics**	**number of patients(%)**
Age, year	36–79
Median age	51
Gender	
Male	15(62.5)
Female	9(37.5)
TNM stage, III + IV	18(75)
Tumor reduction(>2/3)	16(66.7)
Lymph node metastasis, yes	20(83.3)

### Establishment of radioresistant GC cell line RR

**Figure 2:**
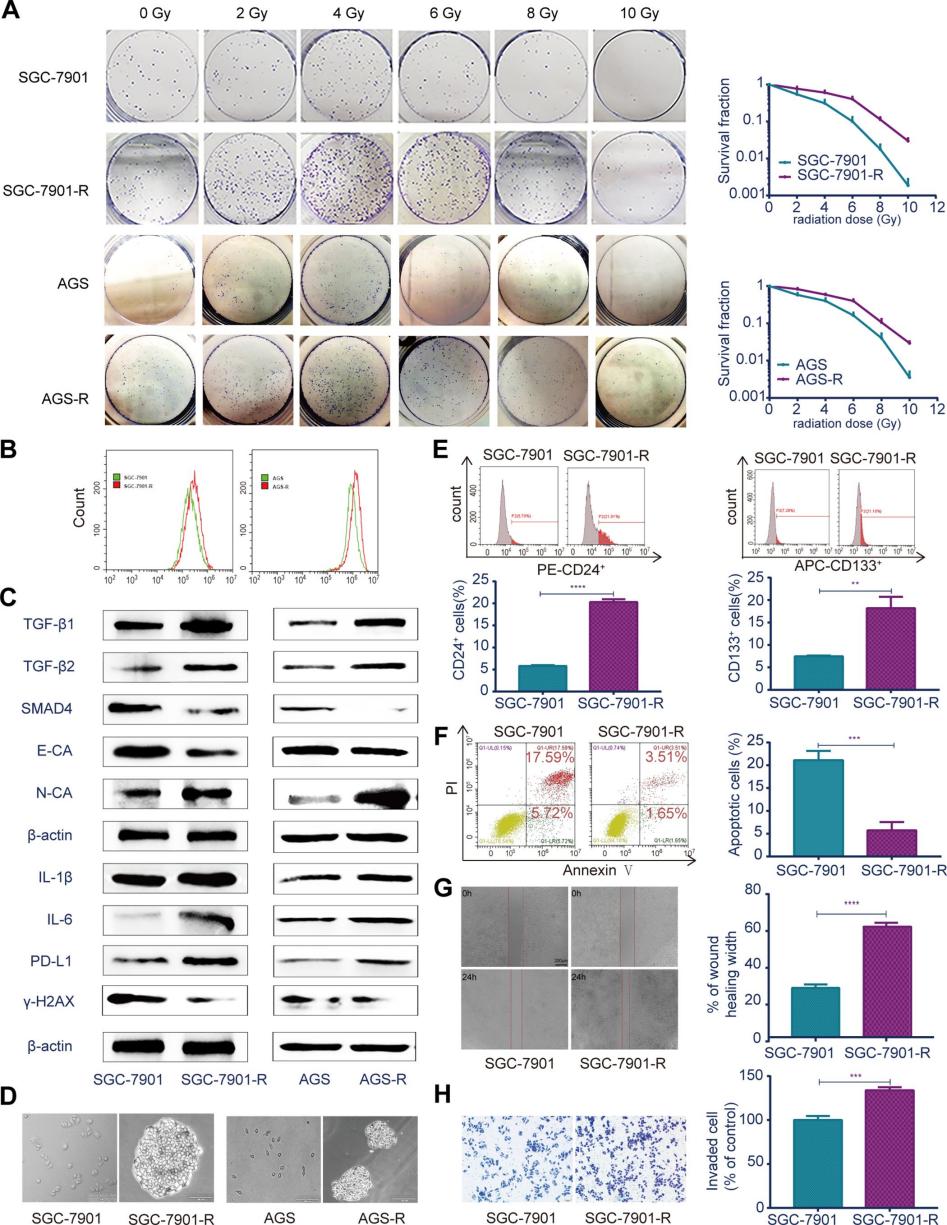
**Different biological characteristics in radioresistant RR and parental GC cells.** (**A**) Clonogenic survival assay: the ability of colony formation between RR and parental GC cells after a range of radiation doses. (**B**) Flow cytometry analysis: ROS levels between RR and parental GC cells. (**C**) Western blot analysis: representative results of the TGF-β1, TGF-β2, SMAD4, E-CA, N-CA, IL-1β, IL-6, PD-L1 and γ-H2AX expressions in RR and parental GC cells. (**D**) Sphere formation assays: captured images of sphere formation assays in RR and parental GC cells. (**E**) Flow cytometry analysis: the expressions of the CSC markers CD24 and CD133 between SGC-7901 and SGC-7901-R cells. (**F**) Flow cytometry analysis: cell apoptosis between SGC-7901 and SGC-7901-R cells after irradiation treatment. (**G**) Cell migration assay: captured images of wound healing assay of SGC-7901 and SGC-7901-R cells, columns indicated the percentage of wound healing width of SGC-7901 and SGC-7901-R cells. (**H**) Cell invasion assay: captured images of transwell assay of SGC-7901 and SGC-7901-R cells, columns indicated the invaded cell percentage of SGC-7901 and SGC-7901-R cells. All data represent three independent experiments, mean ± SEM, **P<0.01, ***P<0.001, ****P<0.0001.

The radioresistant GC cell line RR (SGC-7901-R and AGS-R) were established by exposing parental GC cells to fractioned irradiation for 6 months at a total dose of 60 Gy and validated via colony-formation assays [25]. Colony formation and survival fractions were significantly increased in the RR cells compared with those of the parental GC cells (Figure 2A; *P*<0.05). The reactive oxygen species (ROS) levels in RR cells were significantly increased compared with those of the controls (Figure 2B; *P*<0.05). Real-time PCR (Supplementary Figure 2A) and western blot (Figure 2C) showed that TGF-β1 and TGF-β2 were overexpressed, SMAD4 and E-cadherin were downregulated, and N-cadherin was upregulated in RR cells. In addition, inflammatory factors IL-1β and IL-6 and immune checkpoint PD-L1 were significantly overexpressed, and the DNA double-strand break biomarker, γ-H2AX, was significantly lower in RR cells. The sphere-formation assay showed that RR cells generated sphere cells and showed self-renewal potential compared with the parental GC cells (Figure 2D). Flow cytometry showed that SGC-7901-R cells possessed considerably enhanced expression of the putative CSC markers, CD24 (*P*<0.0001) and CD133 (*P*=0.0018; Figure 2E). Cell apoptosis analysis showed that irradiation induced fewer SGC-7901-R cells into apoptosis (*P*=0.0006; Figure 2F). Wound-healing assays indicated that the wound-healing percentages were significantly increased in SGC-7901-R cells (mean ± SEM: 60.43% ± 0.9563% vs 26.73% ± 1.099%, n=3, *P*<0.0001; Figure 2G). Transwell assays showed that the numbers of invaded cells were significantly increased in SGC-7901-R cells (449 vs 335, n=3, *P*=0.0006; Figure 2H). The same phenotype was also observed in AGS-R cells (Supplementary Figure 2B–2E).

### TGF-β inhibitor LY enhanced GC cell sensitivity to irradiation

**Figure 3:**
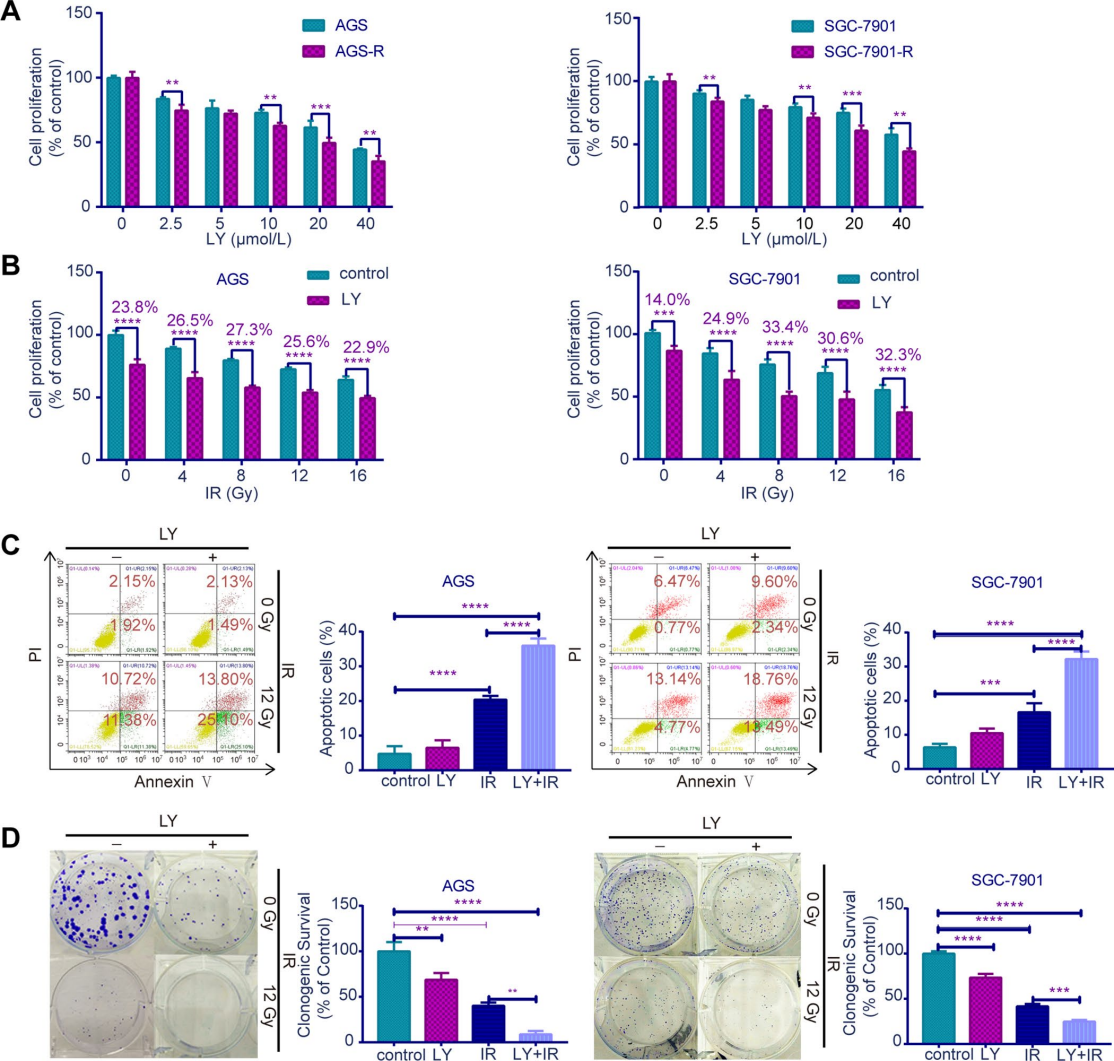
**Effects of LY on radiosensitivity of GC cell lines.** (**A**) CCK-8 assay: the proliferation of RR and parental GC cells influenced by LY. (**B**) CCK-8 assay: the proliferation of GC cells influenced by LY with or without irradiation treatment. (**C**) Flow cytometry analysis: the cell apoptosis in GC cells influenced by LY with or without irradiation treatment. (**D**) Clonogenic survival analysis: the clonogenic ability of GC cells influenced by LY with or without irradiation treatment. All data represent three independent experiments, mean ± SEM, **P<0.01, ***P<0.001, ****P<0.0001.

**Figure 4:**
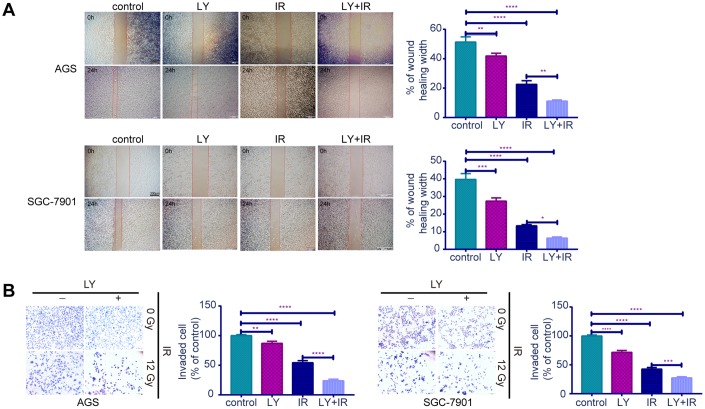
**Effects of LY on the migration and invasion abilities of irradiation induced GC cell lines.** (**A**) Cell migration assay: captured images of wound healing assay in GC cells influenced by LY with or without irradiation treatment, columns indicated the percentage of wound healing width in four groups. (**B**) Cell invasion assay: captured images of transwell assay in GC cells influenced by LY with or without irradiation treatment, columns indicated the invaded cell percentage in four groups. All data represent three independent experiments, mean ± SEM, *P<0.05, **P<0.01, ***P<0.001, ****P<0.0001.

Since TGF-β was upregulated in RR, we examined whether the TGF-β inhibitor LY could lead to radiosensitivity in GC cells. CCK-8 assays (Figure 3A, 3B) indicated that RR clones were more sensitive to LY, and LY pretreatment inhibited GC cell proliferation dose- dependently following irradiation (*P*<0.01). Flow cytometry showed that LY combined with IR increased GC cell apoptosis compared with that of IR alone (*P*<0.0001; Figure 3C). Clonogenic assays showed that LY significantly reduced the clonogenic ability of GC cells when combined with IR (*P*=0.0021 and *P*=0.0005; Figure 3D). The wound-healing (Figure 4A) and transwell assays (Figure 4B) confirmed that LY significantly decreased GC cell migration and invasion abilities after irradiation treatment (*P*<0.05).

### LY inhibited the TGF-β/SMAD4 pathway and altered the expression of other downstream molecules in GC cells

**Figure 5:**
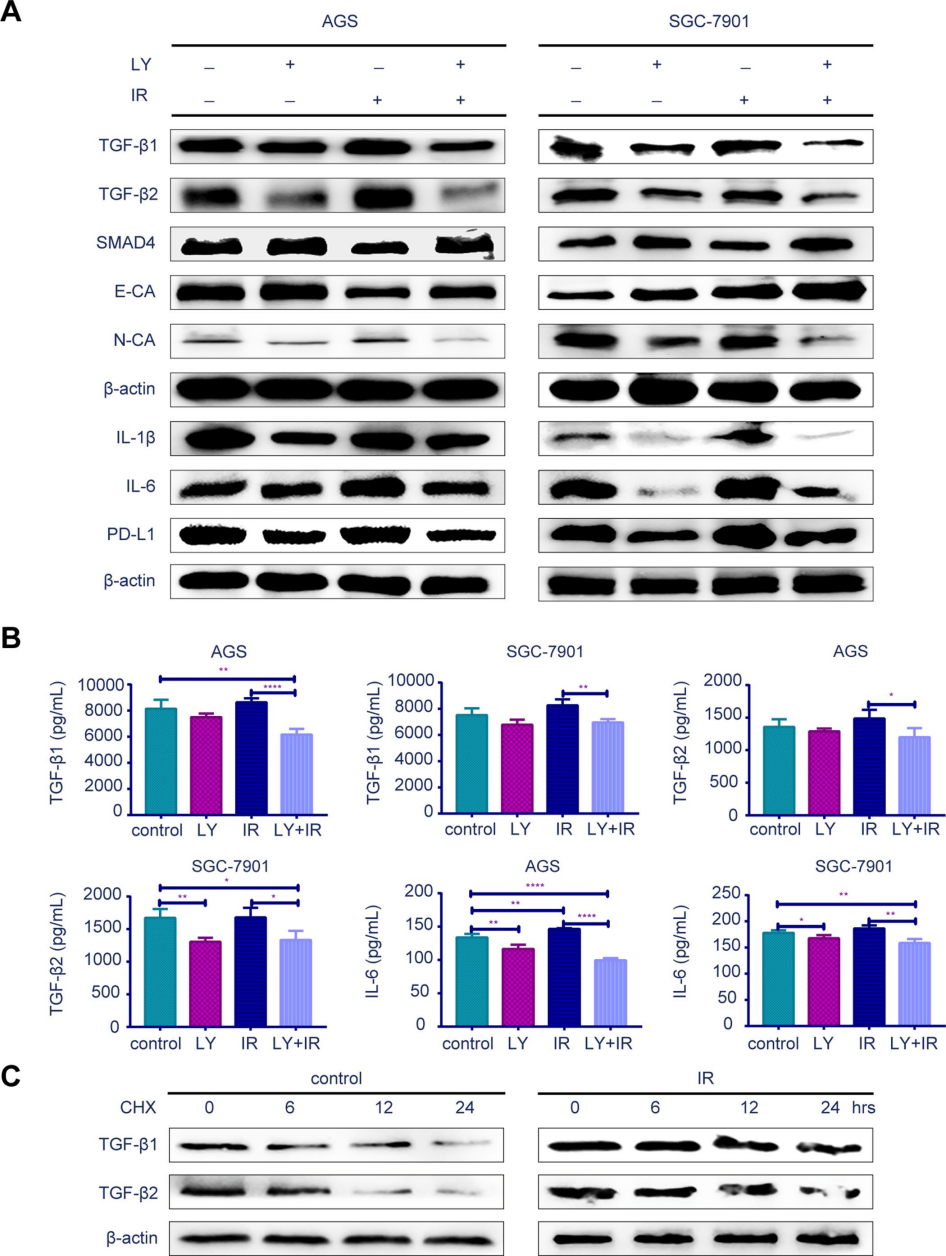
**Effect of LY with or without irradiation on the gene expression of GC cell lines.** (**A**) Western blot analysis: representative results of the TGF-β1, TGF-β2, SMAD4,E-CA, N-CA, IL-1β, IL-6, PD-L1 and γ-H2AX expressions in GC cells after LY with or without irradiation treatment. (**B**) ELISA: the expression of TGF-β1, TGF-β2 and IL-6 in culture medium of GC cells after LY with or without irradiation treatment. (**C**) Western blot analysis: the protein stability of TGF-β1 and TGF-β2 regulated in response to irradiation. All data represent three independent experiments, mean ± SEM, *P<0.05, **P<0.01, ***P<0.001, ****P<0.0001.

**Figure 6:**
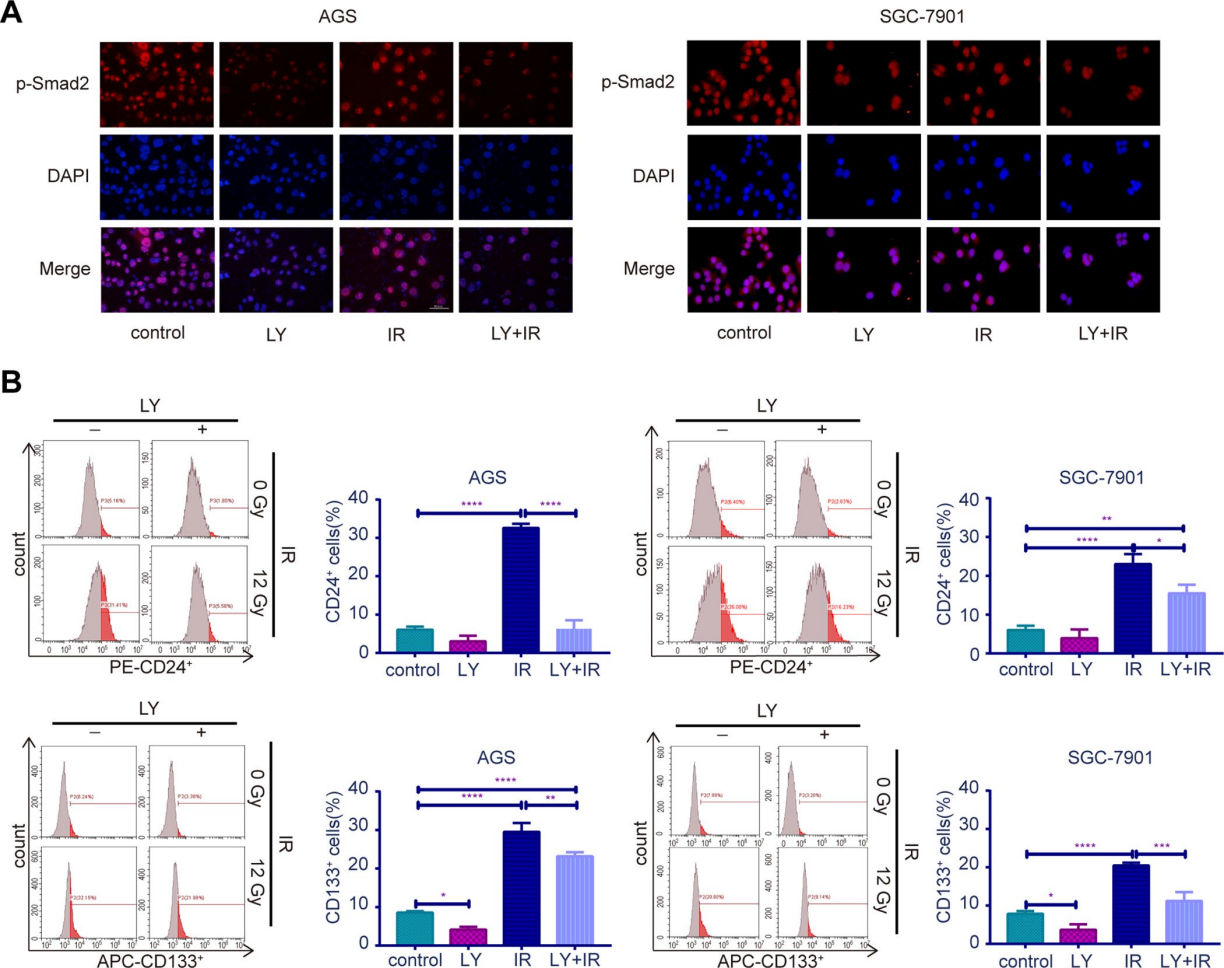
**Effect of LY with or without irradiation on the expression of p-Smad2 and CSC markers in GC cell lines.** (**A**) Immunofluorescence assay: representative results of the p-Smad2 expressions in GC cells after LY with or without irradiation treatment. (**B**) Flow cytometry analysis: the representative results of the expressions of the putative stem cell markers CD24 and CD133 in GC cells with or without LY and irradiation treatment.

To determine the potential mechanism of LY in GC radioresistance, we detected several TGF-β-related signaling pathways in GC cell lines. PCR (Supplementary Figure 2F), western blotting (Figure 5A) and enzyme-linked immunosorbent assay (ELISA; Figure 5B) revealed that LY decreased TGF-β1 and TGF-β2 overexpression after irradiation treatment. LY enhanced SMAD4 expression, reversed the irradiation-induced EMT in GC cells and decreased the expressions of inflammatory-related factors IL-1β and IL-6 and immune checkpoint PD-L1. Pretreating SGC-7901 cells with cycloheximide to inhibit protein synthesis showed that TGF-β protein stability was significantly increased in the IR group compared with that in the control group (Figure 5C). Immunofluorescence images showed that LY inhibited phospho-SMAD2 expression after IR treatment (Figure 6A). Flow cytometry assays showed that LY significantly reduced the overexpression of CSC markers CD24 and CD133 in irradiated GC cell lines (*P*<0.05; Figure 6B and Supplementary Figure 3A).

### LY enhanced the radiosensitivity of a GC xenograft tumor model in vivo

**Figure 7:**
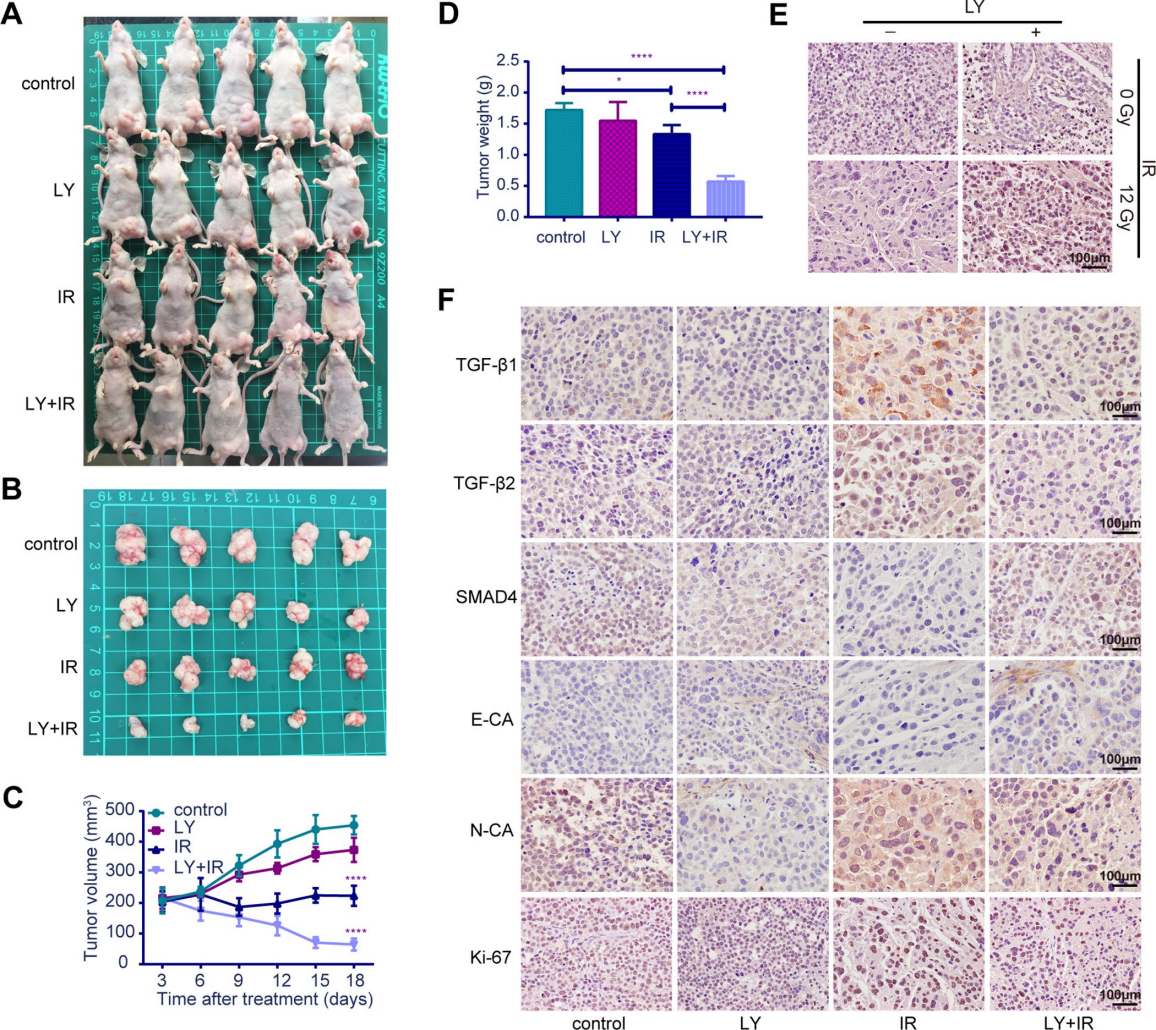
**Effects of LY on radiosensitivity of gastric cancer in vivo.** (**A** and **B**) Changes in tumor sizes: images of BALB/c nude mice with tumors and resected tumors after LY with or without irradiation treatment. (**C**) Changes in tumor volumes: the statistical curves of gastric tumor volumes influenced by LY with or without irradiation treatment. (**D**) Changes in tumor weights: the final tumor weight after LY with or without irradiation treatment for 18 days. (**E**) TUNEL assays: representative images of the apoptostic cells in GC xenografts influenced by LY with or without irradiation treatment. (**F**) Changes in genetic expression in GC xenografts: the immunohistochemical analysis of the TGF-β1, TGF-β2, SMAD4, E-CA, N-CA and Ki-67 expressions in GC xenografts influenced by LY with or without irradiation treatment. Original magnification, ×400. *P<0.05, ****P<0.0001.

To verify the radiosensitization effect of LY on GC in vivo, we established SGC-7901 subcutaneous xenograft tumor models in BALB/c nude mice and examined the effect of LY alone, IR alone, and a combination of LY with IR on the growth of subcutaneous xenograft tumors. The tumor volume was measured twice weekly, and the tumor volumes of the control, LY, and IR-alone groups were larger than those of the LY with IR group (Figures 7A and 7B). Tumor growth was inhibited in mice treated with LY and IR combination (approximately 85% inhibition at day 18) and tumor growth curves were delayed in IR and LY with IR group, (the tumor doubling time of control and LY group was 9 days and 15 days, respectively, and the tumor stopped growing and the tumor volume gradually decreased in IR and LY with IR group 3 days after treatment; *P*<0.0001; Figures 7C). The tumor weight in the LY with IR group was significantly reduced compared with that of the IR group (*P*<0.0001; Figures 7D).

### LY affected the radiation-induced genetic expression in vivo

TUNEL assay indicated that apoptotic cells were increased in the LY with IR group compared with those of the IR-alone group (Figure 7E). The expressions of TGF-β1, TGF-β2, SMAD4, E-cadherin, N-cadherin and Ki-67 in tumor xenografts were detected via immune- histochemical staining. Figure 7F from left to right shows the control, LY-alone, IR-alone and LY with IR groups. TGF-β1, TGF-β2, and N-cadherin expressions were significantly upregulated in tumor xenografts treated with IR alone but were decreased after LY with IR treatment. The expressions of SMAD4 and E-cadherin were significantly downregulated in tumor xenografts with IR treatment alone and were restored in the LY pretreatment group. The LY with IR group showed decreased Ki-67 expression. The staining indices differed statistically (Supplementary Figure 3B). These results were consistent with the in vitro results and confirmed that LY enhanced GC radiosensitivity via TGF-β/SMAD4 signaling pathways and promoted EMT reversal in vivo.

## DISCUSSION

As one of the most common treatments for GC, radiotherapy prolongs patients’ survival and improves their prognoses [26]. However, it also negatively affects the surrounding normal tissues by producing ROS and GC patients continue to develop resistance to radiotherapy [27]. Since increased ROS can influence the tumor microenvironment and activate TGF-β expression, we predicted that high TGF-β expression was related to radioresistance. Our study evidenced that the TGF-β/SMAD4 pathway is activated after radiotherapy for GC, and blocking the TGF-β/SMAD4 pathway with the TGF-β-receptor inhibitor LY can reverse GC radioresistance.

The TGF-β signaling pathway is involved in the tumor microenvironment and influences many cellular processes [28], including cell growth, differentiation and apoptosis, in both tumor and normal tissues. High TGF-β expression is associated with poor survival among cancer patients [29]. Generally, upon binding the TGF-β-type receptor type I and type II, the TGF-β-receptor complex phosphorylates and activates SMAD2 or SMAD3 and forms complexes with SMAD4, then translocates into the nucleus, mediates various gene expressions and affects a range of biological functions in the tumor microenvironment. We found that ROS levels were upregulated in our established radioresistant GC cell line RR and the TGF-β/SMAD4 signaling pathway was activated in radioresistant GC patients and in RR cells. Therefore, ionizing radiation results in the upregulation of ROS then activates the TGF-β signal in radioresistant GC cell lines. In addition, the TGF-β/SMAD4 signaling pathway was activated in the GC cell lines after irradiation exposure, and the TGF-β receptor inhibitor LY increased the radiosensitivity of GC by inhibiting TGF-β/SMAD4 pathway activation both in vitro and in vivo. Invasion and migration abilities are indicators of the degree of malignancy of tumor cells. Some researchers have shown that tumor cells become more aggressive after irradiation treatment [30]. In the present study, the radioresistant GC cell line RR, exhibited stronger invasive and migratory abilities than did the parental GC cell line. AGS and SGC-7901 cells became more invasive and migratory when exposed to irradiation, and LY reduced the increased invasion and migration abilities in this process.

Evidence has shown that EMT is associated with radioresistance [31], and EMT in GC promotes tumor development [32]. This process involves converting epithelial cells into mesenchymal cells in the tumor microenvironment with decreased E-cadherin and increased N-cadherin. Studies have shown that TGF-β is involved in mediating EMT through various mechanisms promoting GC cell migration and invasion and conferring resistance to chemotherapy [33, 34]. As expected, we determined that E-cadherin expression was highly downregulated in radioresistant GC patients and radioresistant GC cell lines accompanied by upregulated N-cadherin. The parental GC cells and GC xenografts exhibited EMT characteristics after irradiation treatment, and LY reversed the EMT in vitro and in vivo to confer radiosensitization.

Inflammation is an important biological marker of cancer in the tumor microenvironment and can influence cellular activities such as proliferation, reproduction and apoptosis [35]. Considerable research has indicated that chronic inflammation contributes to tumor development via cellular mediation of immune cells [36]. IL-1β and IL-6 from the IL-1 cytokine family are important mediators of the inflammatory response and are correlated with GC development [37, 38]. The present results showed an increase in inflammatory cytokines IL-1β and IL-6 in GC cells after irradiation exposure, but LY markedly reduced the inflammatory response and increased radiosensitivity in GC cells. Thus, the TGF-β-receptor inhibitor LY enhanced the radiosensitivity of GC cells by exerting anti-inflammatory effects.

Research has shown that a loss of innate immunity or attenuation of acquired immunity are fundamental to many malignant tumors, including GC [39]. With the mechanism of immune escape, immune cells were regulated via related signaling pathways to exert immunosuppression, thereby affecting tumor cell growth, progression and metastasis in the tumor microenvironment [40]. Research has indicated that immune system inhibition is closely linked to radioresistance in some malignant tumors such as lung cancer [41] and cervical cancer [42], but the association between GC radioresistance and immunosuppression was unclear until now. PD1 and PD-L1 encode the immune-inhibitory ligand and receptor, respectively, and the interaction between those two molecules provides an immune escape for tumor cells through cytotoxic T-cell inactivation to attenuate antitumor immunity in tumors [43]. In the present study, we confirmed that the expression of immune checkpoint inhibition molecule PD-L1 was upregulated in GC cells after irradiation treatment, and LY reduced the immunosuppression by decreasing PD-L1 expression and played an important role in increasing radiosensitivity.

CSCs in the tumor microenvironment are reported to participate in sustaining initiation, propagation, heterogeneity, and self-renewal in tumors and have been confirmed to be closely associated with radioresistance and chemoresistance [44, 45]. Many CSC markers, including CD24, CD44, CD90, CD133, vimentin and aldehyde dehydrogenase 1 (ALDH1), are essential for maintaining stemness properties [46]. In our study, RR cells generated sphere cells and showed self-renewal potential, the CSC markers CD24 and CD133 were upregulated in GC cells when exposed to irradiation, and LY decreased their high expressions. Thus, the radioresistant cell line, RR, and irradiation-treated GC cells exhibit CSC characteristics, and the TGF-β-receptor inhibitor LY reduced CSC marker expression and enhanced the radiosensitivity of GC cell lines to exert its antitumor effect.

Here, the TGF-β/SMAD4 signaling pathway was highly upregulated in radioresistant GC patients and radioresistant GC cell lines. The mechanism by which TGF-β was involved in radioresistance may be determined by affecting the EMT, the cytokine-related signaling pathway, immunocheckpoint and CSC maintenance in GC cells. Inactivation of the TGF-β/SMAD4 signaling pathway with the specific TGF-β-receptor inhibitor LY contributed to the sensitivity of GC both in vitro and in vivo. Our findings suggested that TGF-β levels in GC patients may help predict the response to irradiation treatment, and LY combined with radiotherapy may enable personalized therapeutic strategies.

## MATERIALS AND METHODS

### Patient data sources and clinical patient specimens

TGF-β mRNA expression and related clinical data on 416 GC patients were obtained from TCGA database. Matching analysis was conducted to select 100 GC patients with low TGF-β1 expression, 103 GC patients with high TGF-β1 expression, 102 GC patients with low TGF-β2 expression and 103 GC patients with high TGF-β2 expression. Survival curves were drawn according to the patients’ survival information.

The study population consisted of 24 patients with advanced GC diagnosed at the Department of Radiation and Medical Oncology in Zhongnan Hospital of Wuhan University from 2012 to 2018. The study was conducted in accordance with the recommendations of the Zhongnan Hospital Ethics and Scientific Committee, and written informed consent was received from each patient before tissue acquisition. We determined 8 radioresistant GC patients (no tumor reduction after radiotherapy and no response to radiotherapy) and 16 radiosensitive GC patients (obvious tumor reduction after radiotherapy or complete response to radiotherapy) according to computed tomography imaging results 2 months after radiotherapy. Tissue samples were obtained from gastroscopic diagnostic biopsies before radiotherapy.

### Immunohistochemistry analysis of tumor tissues in GC patients

Immunohistochemistry staining was used to assess gene expression in radioresistant and radiosensitive GC patients. Paraffin-embedded patient tissue sections were subjected to antigen retrieval using 0.01 M citrate buffer (pH 6.0). After blocking the endogenous peroxidase, the tissue sections were incubated with primary antibody overnight in a 4 °C wet box. The antibodies used were as follows: TGF-β1 (#ab179695, Abcam, 1:200), TGF-β2 (#P61812, CUSABIO, 1:100), SMAD4 (#46535, CST, 1:200), E-CA (#3195, CST, 1:200), and N-CA (#13116, CST, 1:200). After washing the sections with phosphate-buffered saline (PBS), horseradish peroxidase (HRP)-labeled secondary antibody was added to incubate at 37 °C for 20 minutes. Color development, counterstaining, dehydration and sealing were performed in sequence, and the results were observed under an optical microscope and judged by three pathologists.

### Cell culture and treatments

The human GC cell lines, AGS and SGC-7901, were purchased from the Type Culture Collection of the Chinese Academy of Sciences (Shanghai, China). The radioresistant GC cell line, RR, was obtained via sublethal dose of irradiation using the Small Animal Radiation Research Platform (PXI X-RAD 225Cx, CT, USA). Cells were cultured in RPMI-1640 (HyClone, USA) supplemented with 10% fetal bovine serum (Gibco, USA) and 1% penicillin-streptomycin sulfate (Invitrogen, USA). TGF-β inhibitor LY2109761 was purchased from Selleckchem (Houston, TX, USA), constituted in dimethyl sulfoxide and stored at -80 °C. All cells were planted and irradiated with 12-Gy X-rays (pretreated with LY 10 μmol/L or not), and cells were exposed to LY 2 h prior to irradiation.

### Establishment and validation of radioresistant GC cell line

The GC cell lines were irradiated with 2-Gy X-rays at 300 cGy/min and an irradiation range of 20 cm × 20 cm to establish the radioresistant GC cell line, RR. The total exposure time was up to 6 months, and the total exposure dose was 60 Gy. After stable passage, a colony-formation assay was used to measure the radioresistance of the RR cells. Cells were seeded into six-well culture plates and exposed to a range of radiation doses (2–10 Gy). Ten days after irradiation, cell colonies were fixed with 4% paraformaldehyde and stained with 0.5% crystal violet. The colonies were counted (up to 50 cells were counted as one colony). The plating efficiency (PE) and survival fraction (SF) were calculated as follows: PE = (colony number/number of inoculated cells) × 100%; SF = colonies counted/(cells seeded × [PE/100]).

### Detection of ROS levels

The collected parental GC cells and radioresistant GC cell lines were incubated with dichlorofluorescein diacetate (DCFH-DA) dye (Beyotime, China) for 25 min at 37 °C, and the ROS levels were detected via flow cytometry (BD Biosciences, USA).

### Sphere formation assays

Sphere-formation assays were conducted to detect GC cell self-renewal. Briefly, 2×10^4^ cells were inoculated in a 10-cm ultra-low-adhesive culture dish, and 10 mL of Dulbecco’s modified Eagle’s medium (DMEM)/F12 containing B27 (1×) and 5 ng/mL epidermal growth factor was added and incubated for 5–7 days to determine spheroid cell growth.

### Cell proliferation assay

A Cell Counting Kit-8 (CCK-8) assay (Dojindo, Japan) was used to measure cell proliferation. GC cells were plated into 96-well plates and treated with an increasing concentration gradient of LY or irradiation (pretreated with LY at 10 μmol/L or not) with a range of radiation doses. After 48 h of incubation, cell viability was measured with CCK-8 following the manufacturer’s instructions. Data are expressed as the percentage of cell proliferation relative to the nonradiated control group and the percentage of cell activity reduced by LY.

### Cell apoptosis assay

Cells were treated as described above. After 24 h of incubation, apoptosis was measured via flow cytometry with 5 μL annexin V and 10 μL propidium iodide cell stains (Multi Sciences, China). Data are expressed as the percentage of apoptotic cells.

### Colony-formation assay

Cells were planted in 6-well plates (600/well). After treatment, the colony-formation ability was detected as described above. Data are expressed as the percentage of the nonradiated control group.

### Cell migration and invasion assessment

Cell migration ability was examined using wound-healing assays. Cells were cultured for 24 h and grown until confluency. Confluent cell monolayers were disrupted by standardized wound scratching with a sterile 200-μL pipette tip, then treated as described above and incubated in 1% FBS medium for 48 h. Cell images of the wound area were captured with an optical microscope (Olympus, Japan). Data are expressed as the percentage of the wound-healing width. Cell invasion ability was detected via transwell assay. At 48 h after treatment, 1 × 10^4^ cells were collected and seeded into the upper chamber, which was precoated with Matrigel (BD Bioscience, USA). Medium containing 10% FBS was added to the lower chamber. After 24 h of incubation, cells that migrated to the lower surface were fixed in methanol, stained and counted under a microscope (Olympus, Japan). Data are expressed as the percentage of the control.

### Flow cytometry assay for putative stem cell markers

Expression of putative stem cell markers CD24 and CD133 in GC cell lines were evaluated by fluorescence-activated cell sorting (FACS) analysis. After treatment, cells were washed with cooled PBS, resuspended, and stained with CD24 and CD133 antibodies (Biolegend, USA). The putative stem cell markers, CD24 and CD133, were detected using a flow cytometer (BD Biosciences, USA). Data are expressed as the percentage of the CD24^+^ or CD133^+^ cells.

### Western blot

Cells were washed with PBS, lysed in 200 μL of protein lysate (containing 100 mg/L phenylmethanesulfonyl fluoride) at 4 °C for 30 min, then the supernatant was centrifuged. The protein was quantified using a BCA Protein Assay kit (Beyotime Institute of Biotechnology, China). Protein (20 μg) was thoroughly mixed with loading buffer, boiled for 5 min, separated by 7.5% sodium dodecyl sulfate-polyacrylamide gel and transferred onto a PVDF membrane (0.22 μm; Millipore). After blocking with 5% skim milk powder for 1 h, the membranes were incubated with the following primary antibodies: TGF-β1 (#ab179695, Abcam, 1:1000), TGF-β2 (#P61812, CUSABIO, 1:600), SMAD4 (#46535, CST, 1:1000), E-CA (#3195, CST, 1:1000), N-CA (#13116, CST, 1:1000), PD-L1 (#MA878942A1m, CUSABIO, 1:600), IL-1β (#PA003023, CUSABIO, 1:600), IL-6 (#PA06757A0Rb, CUSABIO, 1:600), γ-H2AX (#155226, mlbio, 1:600) and β-actin (#60008-1-Ig, Proteintech, 1:5000).

### ELISA

The amounts of TGF-β1, TGF-β2 and IL-6 secreted in the culture medium were detected with ELISA kits (DAKEWE, China) following the manufacturer’s instructions. Data are expressed as the quantity of secreted protein in the culture medium.

### Immunofluorescence

phospho-SMAD2 expressions were detected via immunofluorescence assays. Cells were collected, fixed with 4% paraformaldehyde, permeabilized with Triton X-100 and washed with PBS. Primary antibody (bioss, China) was added and incubated in a wet box at 37 °C for 2 h. Secondary antibodies to the corresponding species of the primary antibody were added and incubated for 50 min in the dark. The cells were stained with DAPI for 5 min, then viewed under an inverted fluorescence microscope to observe and collect images.

### Animal model establishment and treatment

Four- to six-week-old BALB/c male nude mice were fed at the animal experiment center of Zhongnan Hospital, and all procedures were approved by the Animal Care and Use Committee of Wuhan University. A total of 1×10^6^ SGC-7901 cells were injected subcutaneously in the left hind limbs of the mice. Twenty mice were randomly divided into 4 groups: the control, LY, IR, and LY plus IR at n=5/group. Treatments started after 20 days when the tumors had reached at least 200 mm^3^. Tumors in the IR and LY plus IR groups were irradiated with fractionated radiotherapy (4×6 Gy, twice weekly); the LY and LY plus IR groups were treated with LY orally twice daily (50 mg/kg, on days 1–5 each week) until the animals were sacrificed. Tumor volume was directly measured twice weekly using a Vernier caliper (volume=length × width × width × 0.5).

### TUNEL assays and tumor immunohistochemistry in the mice

All animals were sacrificed via carbon dioxide inhalation after 18 days of treatment, and the tumor tissues were removed and measured. The tissues were sectioned, TUNEL assays (Roche, China) were used to detect cell apoptosis in vivo following the manufacturer’s instructions, and gene expression at the protein level was detected via immunohistochemistry as described above.

### Statistical analysis

Each experiment was performed at least three times to ensure the repeatability of the results. Statistical analysis was performed using GraphPad Prism 7.0 (GraphPad Software). The results were analyzed using Student’s t-test when two groups were compared or one-way analysis of variance when more than two groups were compared. OS curves were plotted using the Kaplan-Meier method and compared using a log-rank test. Differences were considered statistically significant at *P*<0.05.

### Ethics approval

The study was conducted in accordance with the recommendations of Zhongnan Hospital Ethics and Scientific Committee and written informed consent was received from each patient before tissue acquisition (Ethical approval number: 2019165). All the procedures in animal experiments were approved by the Animal Care and Use Committee of Wuhan University.

## Supplementary Material

Supplementary Figures

## References

[1] Xu W, Yang Z, Lu N. Molecular targeted therapy for the treatment of gastric cancer. J Exp Clin Cancer Res. 2016; 35:1. 10.1186/s13046-015-0276-9 26728266 PMC4700735

[2] Zhang X, Zheng L, Sun Y, Wang T, Wang B. Tangeretin enhances radiosensitivity and inhibits the radiation-induced epithelial-mesenchymal transition of gastric cancer cells. Oncol Rep. 2015; 34:302–10. 10.3892/or.2015.3982 25998143

[3] Jung C, Motwani M, Kortmansky J, Sirotnak FM, She Y, Gonen M, Haimovitz-Friedman A, Schwartz GK. The cyclin-dependent kinase inhibitor flavopiridol potentiates gamma-irradiation-induced apoptosis in colon and gastric cancer cells. Clin Cancer Res. 2003; 9:6052–61. 14676132

[4] Chen L, Yuan D, Yang Y, Ren M. LincRNA-p21 enhances the sensitivity of radiotherapy for gastric cancer by targeting the β-catenin signaling pathway. J Cell Biochem. 2019; 120:6178–87. 10.1002/jcb.27905 30484893

[5] Núñez FJ, Mendez FM, Kadiyala P, Alghamri MS, Savelieff MG, Garcia-Fabiani MB, Haase S, Koschmann C, Calinescu AA, Kamran N, Saxena M, Patel R, Carney S, et al. IDH1-R132H acts as a tumor suppressor in glioma via epigenetic up-regulation of the DNA damage response. Sci Transl Med. 2019; 11. 10.1126/scitranslmed.aaq1427 30760578 PMC6400220

[6] Bernichon E, Vallard A, Wang Q, Attignon V, Pissaloux D, Bachelot T, Heudel PE, Ray-Coquard I, Bonnet E, de la Fouchardière A, Faure C, Chopin N, Beurrier F, et al. Genomic alterations and radioresistance in breast cancer: an analysis of the ProfiLER protocol. Ann Oncol. 2017; 28:2773–79. 10.1093/annonc/mdx488 28945826

[7] Todorova PK, Fletcher-Sananikone E, Mukherjee B, Kollipara R, Vemireddy V, Xie XJ, Guida PM, Story MD, Hatanpaa K, Habib AA, Kittler R, Bachoo R, Hromas R, et al. Radiation-induced DNA damage cooperates with heterozygosity of TP53 and PTEN to generate high grade gliomas. Cancer Res. 2019; 79:3749–61. 10.1158/0008-5472.CAN-19-0680 31088835 PMC6635038

[8] Wang XC, Yue X, Zhang RX, Liu TY, Pan ZZ, Yang MJ, Lu ZH, Wang ZY, Peng JH, Le LY, Wang GY, Peng QH, Meng Y, et al. Genome-wide RNAi screening identifies RFC4 as a factor that mediates radioresistance in colorectal cancer by facilitating non-homologous end joining repair. Clin Cancer Res. 2019; 25:4567–79. 10.1158/1078-0432.ccr-18-3735 30979744

[9] McGee HM, Jiang D, Soto-Pantoja DR, Nevler A, Giaccia AJ, Woodward WA. Targeting the Tumor Microenvironment in Radiation Oncology: Proceedings from the 2018 ASTRO-AACR Research Workshop. Clin Cancer Res. 2019; 25:2969–2974. 10.1158/1078-0432.CCR-18-3781 30723144 PMC7265991

[10] Stapleton S, Jaffray D, Milosevic M. Radiation effects on the tumor microenvironment: implications for nanomedicine delivery. Adv Drug Deliv Rev. 2017; 109:119–30. 10.1016/j.addr.2016.05.021 27262923

[11] Gomez-Casal R, Bhattacharya C, Ganesh N, Bailey L, Basse P, Gibson M, Epperly M, Levina V. Non-small cell lung cancer cells survived ionizing radiation treatment display cancer stem cell and epithelial-mesenchymal transition phenotypes. Mol Cancer. 2013; 12:94. 10.1186/1476-4598-12-94 23947765 PMC3751356

[12] Pan Y, Zhou F, Zhang R, Claret FX. Stat3 inhibitor Stattic exhibits potent antitumor activity and induces chemo- and radio-sensitivity in nasopharyngeal carcinoma. PLoS One. 2013; 8:e54565. 10.1371/journal.pone.0054565 23382914 PMC3558509

[13] Tabraue C, Lara PC, De Mirecki-Garrido M, De La Rosa JV, López-Blanco F, Fernández-Pérez L, Boscá L, Castrillo A. LXR Signaling Regulates Macrophage Survival and Inflammation in Response to Ionizing Radiation. Int J Radiat Oncol Biol Phys. 2019; 104:913–23. 10.1016/j.ijrobp.2019.03.028 30922944

[14] Ilkow CS, Marguerie M, Batenchuk C, Mayer J, Ben Neriah D, Cousineau S, Falls T, Jennings VA, Boileau M, Bellamy D, Bastin D, de Souza CT, Alkayyal A, et al. Reciprocal cellular cross-talk within the tumor microenvironment promotes oncolytic virus activity. Nat Med. 2015; 21:530–36. 10.1038/nm.3848 25894825

[15] Qiao C, Yang J, Shen Q, Liu R, Li Y, Shi Y, Chen J, Shen Y, Xiao Z, Weng J, Zhang X. Traceable Nanoparticles with Dual Targeting and ROS Response for RNAi-Based Immunochemotherapy of Intracranial Glioblastoma Treatment. Adv Mater. 2018; 30:e1705054. 10.1002/adma.201705054 29577457

[16] Li QL, Ito K, Sakakura C, Fukamachi H, Inoue K, Chi XZ, Lee KY, Nomura S, Lee CW, Han SB, Kim HM, Kim WJ, Yamamoto H, et al. Causal relationship between the loss of RUNX3 expression and gastric cancer. Cell. 2002; 109:113–24. 10.1016/S0092-8674(02)00690-6 11955451

[17] Tyekucheva S, Bowden M, Bango C, Giunchi F, Huang Y, Zhou C, Bondi A, Lis R, Van Hemelrijck M, Andrén O, Andersson SO, Watson RW, Pennington S, et al. Stromal and epithelial transcriptional map of initiation progression and metastatic potential of human prostate cancer. Nat Commun. 2017; 8:420. 10.1038/s41467-017-00460-4 28871082 PMC5583238

[18] Chiba N, Comaills V, Shiotani B, Takahashi F, Shimada T, Tajima K, Winokur D, Hayashida T, Willers H, Brachtel E, Vivanco MD, Haber DA, Zou L, Maheswaran S. Homeobox B9 induces epithelial-to-mesenchymal transition-associated radioresistance by accelerating DNA damage responses. Proc Natl Acad Sci U S A. 2012; 109:2760–5. 10.1073/pnas.1018867108 21930940 PMC3286905

[19] Qiao P, Li G, Bi W, Yang L, Yao L, Wu D. microRNA-34a inhibits epithelial mesenchymal transition in human cholangiocarcinoma by targeting Smad4 through transforming growth factor-beta/Smad pathway. BMC Cancer. 2015; 15:469. 10.1186/s12885-015-1359-x 26077733 PMC4477414

[20] Chen X, Wang L, Li P, Song M, Qin G, Gao Q, Zhang Z, Yue D, Wang D, Nan S, Qi Y, Li F, Yang L, et al. Dual TGF-β and PD-1 blockade synergistically enhances MAGE-A3-specific CD8^+^ T cell response in esophageal squamous cell carcinoma. Int J Cancer. 2018; 143:2561–74. 10.1002/ijc.31730 29981155

[21] Ma YM, Sun T, Liu YX, Zhao N, Gu Q, Zhang DF, Qie S, Ni CS, Liu Y, Sun BC. A pilot study on acute inflammation and cancer: a new balance between IFN-gamma and TGF-beta in melanoma. J Exp Clin Cancer Res. 2009; 28:23. 10.1186/1756-9966-28-23 19228418 PMC2683570

[22] Zhang M, Kleber S, Röhrich M, Timke C, Han N, Tuettenberg J, Martin-Villalba A, Debus J, Peschke P, Wirkner U, Lahn M, Huber PE. Blockade of TGF-beta signaling by the TGFbetaR-I kinase inhibitor LY2109761 enhances radiation response and prolongs survival in glioblastoma. Cancer Res. 2011; 71:7155–67. 10.1158/0008-5472.CAN-11-1212 22006998

[23] Bu JQ, Chen F. TGF-β1 promotes cells invasion and migration by inducing epithelial mesenchymal transformation in oral squamous cell carcinoma. Eur Rev Med Pharmacol Sci. 2017; 21:2137–44. 28537671

[24] Siu MK, Tsai YC, Chang YS, Yin JJ, Suau F, Chen WY, Liu YN. Transforming growth factor-β promotes prostate bone metastasis through induction of microRNA-96 and activation of the mTOR pathway. Oncogene. 2015; 34:4767–76. 10.1038/onc.2014.414 25531317

[25] Feng XP, Yi H, Li MY, Li XH, Yi B, Zhang PF, Li C, Peng F, Tang CE, Li JL, Chen ZC, Xiao ZQ. Identification of biomarkers for predicting nasopharyngeal carcinoma response to radiotherapy by proteomics. Cancer Res. 2010; 70:3450–62. 10.1158/0008-5472.CAN-09-4099 20406978

[26] Yi H, Yan X, Luo Q, Yuan L, Li B, Pan W, Zhang L, Chen H, Wang J, Zhang Y, Zhai Y, Qiu MZ, Yang DJ. A novel small molecule inhibitor of MDM2-p53 (APG-115) enhances radiosensitivity of gastric adenocarcinoma. J Exp Clin Cancer Res. 2018; 37:97. 10.1186/s13046-018-0765-8 29716622 PMC5930807

[27] Gu H, Huang T, Shen Y, Liu Y, Zhou F, Jin Y, Sattar H, Wei Y. Reactive Oxygen Species-Mediated Tumor Microenvironment Transformation: The Mechanism of Radioresistant Gastric Cancer. Oxid Med Cell Longev. 2018; 2018:5801209. 10.1155/2018/5801209 29770167 PMC5892229

[28] Zhang F, Li F, Lu GH, Nie W, Zhang L, Lv Y, Bao W, Gao X, Wei W, Pu K, Xie HY. Engineering Magnetosomes for Ferroptosis/Immunomodulation Synergism in Cancer. ACS Nano. 2019; 13:5662–5673. 10.1021/acsnano.9b00892 31046234

[29] Avgustinova A, Iravani M, Robertson D, Fearns A, Gao Q, Klingbeil P, Hanby AM, Speirs V, Sahai E, Calvo F, Isacke CM. Tumour cell-derived Wnt7a recruits and activates fibroblasts to promote tumour aggressiveness. Nat Commun. 2016; 7:10305. 10.1038/ncomms10305 26777421 PMC4735631

[30] Wozny AS, Vares G, Alphonse G, Lauret A, Monini C, Magné N, Cuerq C, Fujimori A, Monboisse JC, Beuve M, Nakajima T, Rodriguez-Lafrasse C. ROS Production and Distribution: A New Paradigm to Explain the Differential Effects of X-ray and Carbon Ion Irradiation on Cancer Stem Cell Migration and Invasion. Cancers (Basel). 2019; 11. 10.3390/cancers11040468 30987217 PMC6521340

[31] Luo M, Wu C, Guo E, Peng S, Zhang L, Sun W, Liu D, Hu G, Hu G. FOXO3a knockdown promotes radioresistance in nasopharyngeal carcinoma by inducing epithelial-mesenchymal transition and the Wnt/β-catenin signaling pathway. Cancer Lett. 2019; 455:26–35. 10.1016/j.canlet.2019.04.019 31022422

[32] Song YX, Sun JX, Zhao JH, Yang YC, Shi JX, Wu ZH, Chen XW, Gao P, Miao ZF, Wang ZN. Non-coding RNAs participate in the regulatory network of CLDN4 via ceRNA mediated miRNA evasion. Nat Commun. 2017; 8:289. 10.1038/s41467-017-00304-1 28819095 PMC5561086

[33] Li F, Shi J, Xu Z, Yao X, Mou T, Yu J, Liu H, Li G. S100A4-MYH9 Axis Promote Migration and Invasion of Gastric Cancer Cells by Inducing TGF-β-Mediated Epithelial-Mesenchymal Transition. J Cancer. 2018; 9:3839–49. 10.7150/jca.25469 30410586 PMC6218764

[34] Zhang H, Ren L, Ding Y, Li F, Chen X, Ouyang Y, Zhang Y, Zhang D. Hyaluronan-mediated motility receptor confers resistance to chemotherapy *via* TGFβ/Smad2-induced epithelial-mesenchymal transition in gastric cancer. FASEB J. 2019; 33:6365–77. 10.1096/fj.201802186R 30802150

[35] Conti A, Gulì C, La Torre D, Tomasello C, Angileri FF, Aguennouz M. Role of inflammation and oxidative stress mediators in gliomas. Cancers (Basel). 2010; 2:693–712. 10.3390/cancers2020693 24281089 PMC3835099

[36] Zhang X, Shi H, Yuan X, Jiang P, Qian H, Xu W. Tumor-derived exosomes induce N2 polarization of neutrophils to promote gastric cancer cell migration. Mol Cancer. 2018; 17:146. 10.1186/s12943-018-0898-6 30292233 PMC6174070

[37] Sliter DA, Martinez J, Hao L, Chen X, Sun N, Fischer TD, Burman JL, Li Y, Zhang Z, Narendra DP, Cai H, Borsche M, Klein C, Youle RJ. Parkin and PINK1 mitigate STING-induced inflammation. Nature. 2018; 561:258–62. 10.1038/s41586-018-0448-9 30135585 PMC7362342

[38] Matak P, Heinis M, Mathieu JR, Corriden R, Cuvellier S, Delga S, Mounier R, Rouquette A, Raymond J, Lamarque D, Emile JF, Nizet V, Touati E, Peyssonnaux C. Myeloid HIF-1 is protective in Helicobacter pylori-mediated gastritis. J Immunol. 2015; 194:3259–66. 10.4049/jimmunol.1401260 25710915

[39] Coleman OI, Haller D. Bacterial Signaling at the Intestinal Epithelial Interface in Inflammation and Cancer. Front Immunol. 2018; 8:1927. 10.3389/fimmu.2017.01927 29354132 PMC5760496

[40] Deng C, Zhang Q, Jia M, Zhao J, Sun X, Gong T, Zhang Z. Tumors and Their Microenvironment Dual-Targeting Chemotherapy with Local Immune Adjuvant Therapy for Effective Antitumor Immunity against Breast Cancer. Adv Sci (Weinh). 2019; 6:1801868. 10.1002/advs.201801868 30937266 PMC6425447

[41] Yang HJ, Kim N, Seong KM, Youn H, Youn B. Investigation of radiation-induced transcriptome profile of radioresistant non-small cell lung cancer A549 cells using RNA-seq. PLoS One. 2013; 8:e59319. 10.1371/journal.pone.0059319 23533613 PMC3606344

[42] Zhang D, Dong Y, Zhao Y, Zhou C, Qian Y, Hegde ML, Wang H, Han S. Sinomenine hydrochloride sensitizes cervical cancer cells to ionizing radiation by impairing DNA damage response. Oncol Rep. 2018; 40:2886–95. 10.3892/or.2018.6693 30226618 PMC6151895

[43] Gong X, Li X, Jiang T, Xie H, Zhu Z, Zhou F, Zhou C. Combined Radiotherapy and Anti-PD-L1 Antibody Synergistically Enhances Antitumor Effect in Non-Small Cell Lung Cancer. J Thorac Oncol. 2017; 12:1085–97. 10.1016/j.jtho.2017.04.014 28478231

[44] Martin-Padura I, Marighetti P, Agliano A, Colombo F, Larzabal L, Redrado M, Bleau AM, Prior C, Bertolini F, Calvo A. Residual dormant cancer stem-cell foci are responsible for tumor relapse after antiangiogenic metronomic therapy in hepatocellular carcinoma xenografts. Lab Invest. 2012; 92:952–66. 10.1038/labinvest.2012.65 22546866

[45] Lin JC, Tsai JT, Chao TY, Ma HI, Chien CS, Liu WH. MSI1 associates glioblastoma radioresistance via homologous recombination repair, tumor invasion and cancer stem-like cell properties. Radiother Oncol. 2018; 129:352–63. 10.1016/j.radonc.2018.09.014 30322656

[46] Zhang J, Cai H, Sun L, Zhan P, Chen M, Zhang F, Ran Y, Wan J. LGR5, a novel functional glioma stem cell marker, promotes EMT by activating the Wnt/β-catenin pathway and predicts poor survival of glioma patients. J Exp Clin Cancer Res. 2018; 37:225. 10.1186/s13046-018-0864-6 30208924 PMC6136228

