# Supplementary material for: TGF-β receptor inhibitor LY2109761 enhances the radiosensitivity of gastric cancer by inactivating the TGF-β/SMAD4 signaling pathway
Source: Aging (Albany NY). 2019 Oct 19;11(20):8892–910. doi: 10.18632/aging.102329 (PMC6834415; doi:10.18632/aging.102329)
Supplement: Supplementary Figures [file aging-11-102329-s001.pdf]

SUPPLEMENTARY FIGURES

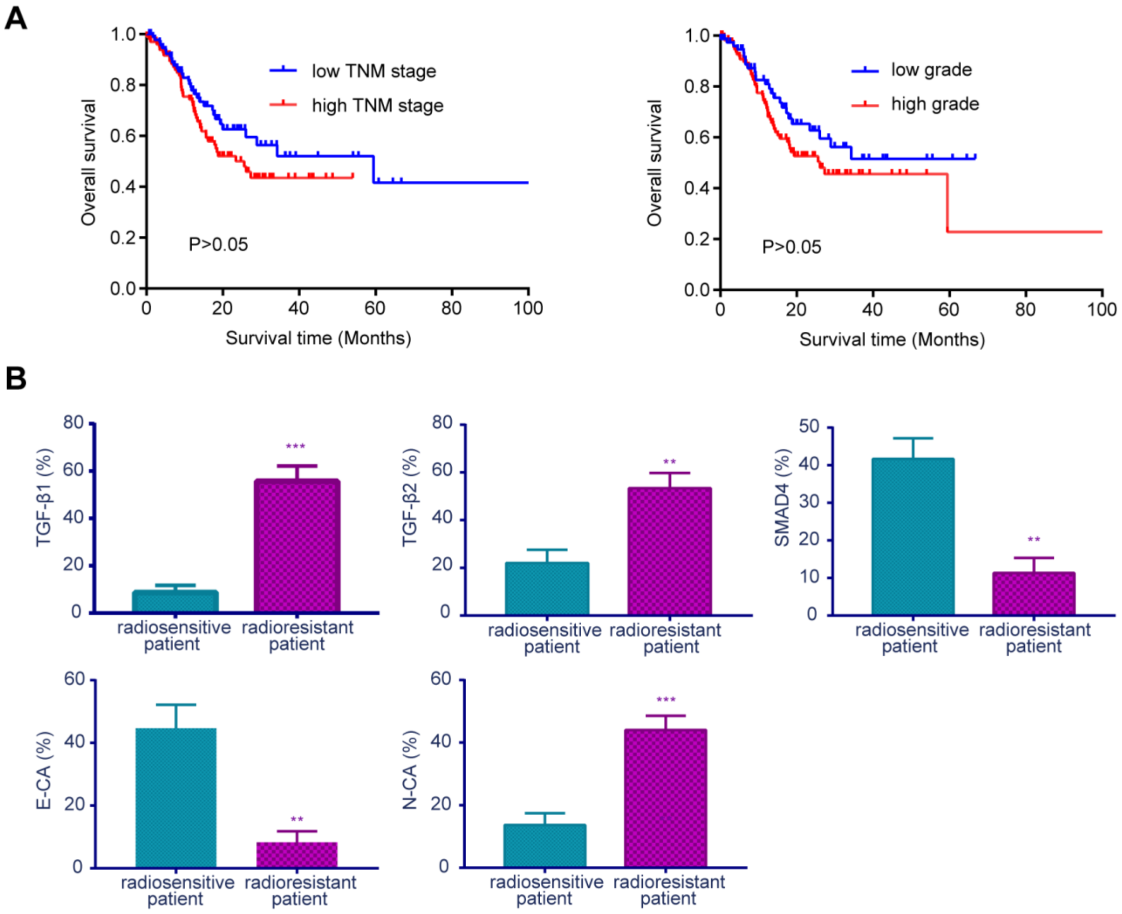

Supplementary Figure 1. (**A**) The relationship between the TNM stage and grade with the overall survival in GC patients. (**B**) The immunohistochemistry staining index of related gene in radiosensitive and radioresistant GC patients.

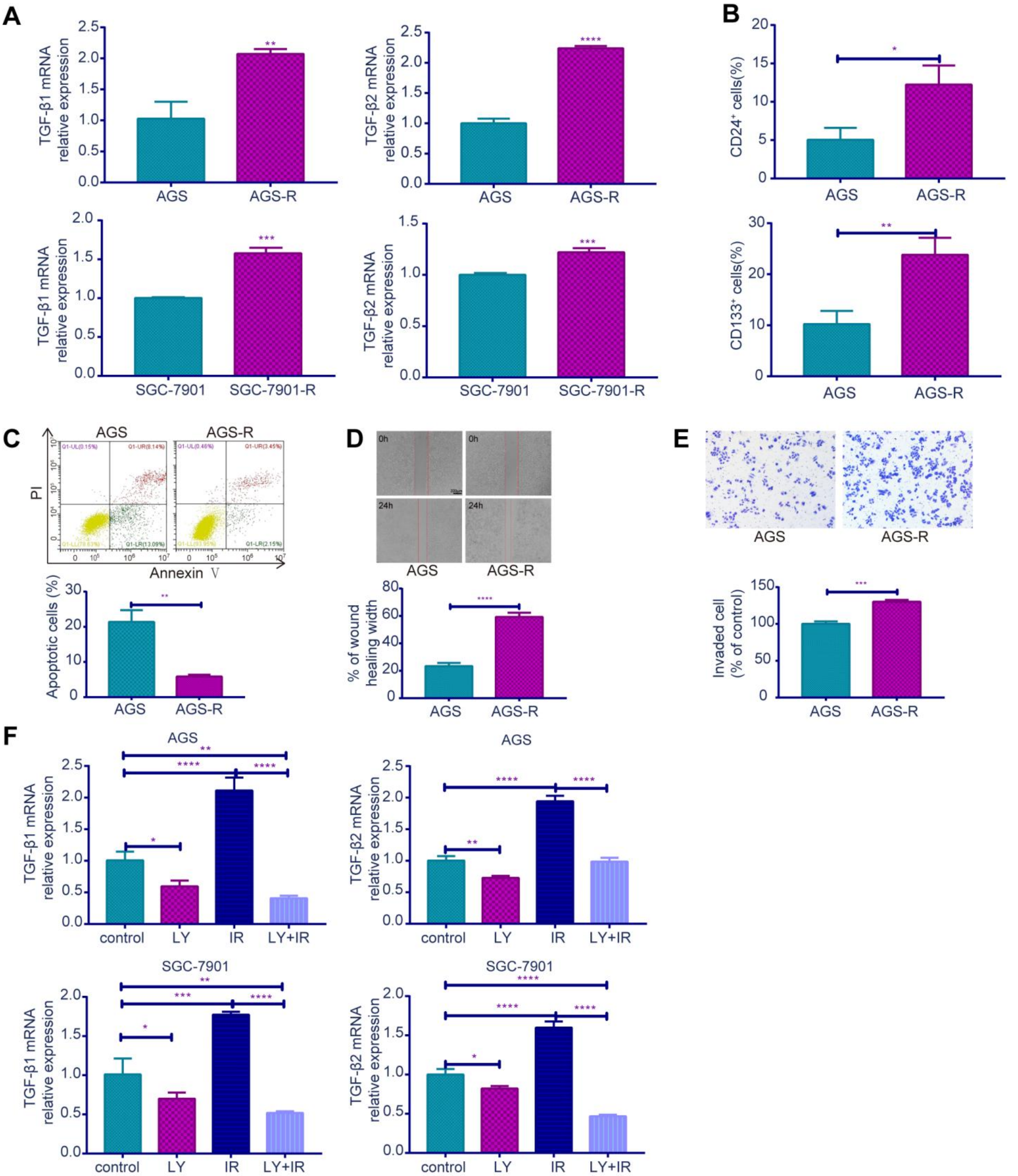

Supplementary Figure 2. (**A**) The TGF-β1 and TGF-β2 expressions in parental GC cells and radioresistant GC cells detected by PCR analysis. (**B**) the expressions of the CSC markers CD24 and CD133 between AGS and AGS-R cells. (**C**) Flow cytometry analysis: cell apoptosis between AGS and AGS-R cells after irradiation treatment. (**D**) Cell migration assay: captured images of wound healing assay of AGS and AGS-R cells, columns indicated the percentage of wound healing width of AGS and AGS-R cells. (**E**) Cell invasion assay: captured images of transwell assay of AGS and AGS-R cells, columns indicated the invaded cell percentage of AGS and AGS-R cells. (**F**) LY inhibited the TGF-β1 and TGF-β2 expressions in irradiated GC cells.

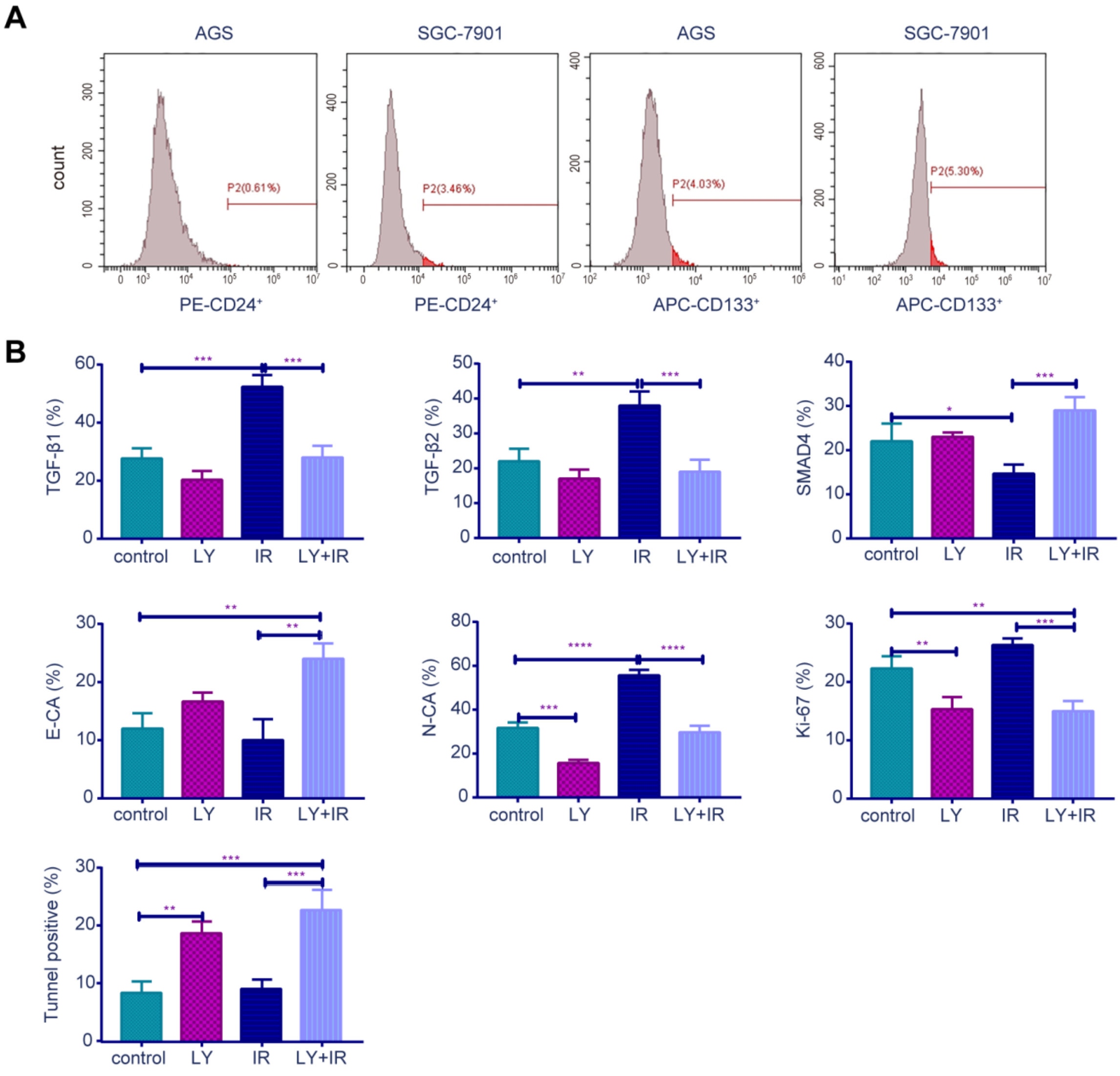

Supplementary Figure 3. (**A**) the representative results of the expressions of putative stem cell markers CD24 and CD133 in GC cells (isotype controls). (**B**) The immunohistochemistry staining index of related gene in GC xenografts.
